# Methodology for Precision Land Use Mapping towards Sustainable Urbanized Land Development

**DOI:** 10.3390/ijerph19063633

**Published:** 2022-03-18

**Authors:** Patrycja Szarek-Iwaniuk, Agnieszka Dawidowicz, Adam Senetra

**Affiliations:** Institute of Spatial Management and Geography, Faculty of Geoengineering, University of Warmia and Mazury in Olsztyn, 10-719 Olsztyn, Poland; patrycja.szarek@uwm.edu.pl (P.S.-I.); agnieszka.dawidowicz@uwm.edu.pl (A.D.)

**Keywords:** land-use/land cover, interpolation, spatial analysis, GIS, land-use mapping, urban planning, field inventory, land-use types, interpolation

## Abstract

Land-use/land cover maps constitute one of the key sources of information on urban space. To address the problems associated with the lack of timely and detailed land-use maps, the authors have developed a universal methodological approach for monitoring land use structure that is particularly useful in a rapidly evolving urban environment. Therefore, the main aim of this study was to develop a universal methodology for high-precision land-use analysis in urbanized areas in the context of large-scale mapping. The method uses geoinformation tools, photogrammetric data (orthophoto maps) as well as data acquired during a field inventory (involving a field survey and field mapping). The proposed approach is based on the modified existing approaches towards a detailed identification of land-use patterns while reducing the difficulties arising from the limitations of existing land use data sources. The methodology consists of several steps. First, the data sources for land-use analysis were selected. Subsequently, the classification of land-use categories in urban space was made. Finally, the method to high-precision land-use analysis for large-scale mapping was defined under the assumption that it is to be universal for use in countries with different levels of spatial and economic development. The proposed research method is based on an interpolation algorithm. It is highly valid, flexible, modifiable, accurate, and it can be applied to process publicly available and free sources of spatial data. Validation of the method on a test object (city of Ostróda, Poland) showed its high effectiveness, which is limited only by the type of data. The results obtained with the use of the proposed method not only supported the determination of the present land-use structure in the town but were also used to identify areas with the highest and lowest intensity and concentration of specific land-cover types.

## 1. Introduction

Urban space undergoes constant changes. It is generally assumed that spatial planning influences the quality of life and the well-being of the community [1,2]. The dynamic of changes in the urban fabric, in particular progressing urban decentralization, contributes to composite urban development [3]. In view of the rapid evolution of the functional structure of cities and dynamic urbanization, there is an urgent need for effective and accurate monitoring of land use in urban space. Precise recognition of land-use patterns and the identification of large-scale spatial changes (for example, in small and medium-sized towns, residential areas, suburban areas, districts and housing estates visualized on a scale of 1:1000, 1:500, and larger) supports planning and the creation of space that is adapted to the needs of local inhabitants and users [4]. Large-scale surveys are surveys with significant proximity detail, which is needed to analyze built-up areas. Large-scale analyses of spatial development enable the determination of functional areas and the influence of anthropogenic pressure on the environment, evaluations of the effect of spatial development on the quality of local life and the identification of areas subjected to urban pressure [5,6,7]. Knowledge of the land-use structure is required to plan remedial actions and eliminate spatial conflicts [8,9]. Therefore, a universal approach involving accurate methods for monitoring changes in a rapidly evolving urban environment is required as an effective analytical tool that relies on available and up-to-date spatial data sources in the planning process.

Research studies investigating land-use structure with the use of various methodologies, tools and database configurations are conducted around the world (comprehensive description in Section 3.1.1 and Section 3.1.2). Such analyses focus on advanced urbanization processes that are the main drivers of spatial change and land-use structure, not only in small and medium-sized towns and large cities but also in suburban and rural areas [10,11,12,13,14,15,16]. However, they have some limitations, including difficulties in ensuring repeatability of surveys, the necessity of high costs (data acquisition, software, etc.), limitations resulting from availability, coverage, and timeliness of source data, as well as limitations resulting from inadequate, too low precision and detail needed to generate accurate maps presenting land use structure of urbanized areas.

To address the problems associated with the lack of timely and detailed land-use maps, the authors have proposed a universal methodological approach for monitoring land use structure. Thus, the main aim of this study was to develop a universal methodology for high-precision land-use analysis in urbanized areas in the context of large-scale mapping. The method uses geoinformation tools, photogrammetric data (orthophoto maps) as well as data acquired during a field inventory (involving a field survey and field mapping). The method was tested in a case study conducted in the Polish town of Ostróda.

A research methodology for generating up-to-date, accurate, and customized land-use maps based on an interpolation algorithm, widely available photogrammetric data, and field inventory data is a precise, universal and useful tool for diagnosing spatial development, in particular in the context of large-scale mapping. The proposed approach has a number of practical implications, which provide universality but also bring novelty to existing land use structure studies. Firstly, it offers a quantitative method for high-precision analysis of land-use structure, including the distribution and concentration of various types of land use in urban space. The described method was applied to generate a land-use map depicting the distribution and the actual location of different land-use types in the studied area, as well as maps presenting the intensity and concentration of various land-use types in the studied area in a continuous manner. Thus, the proposed method offers two approaches to visualizing the studied phenomenon, which is an advantage over other research methods for analyzing land-use structure.

Secondly, it is a universal method that can be applied in countries with different levels of spatial, economic and technological development. Based on the proposed data sources, the method can be used globally, including in countries where other sources of data are available, as well as in countries where such data cannot be acquired. The sources of spatial data used in the proposed research method are widely available, free of charge and up-to-date, which provides an advantage over methods that rely on data sources that are available for a fee or have limited spatial coverage. In addition, the use of the proposed data sources ensures the highest validity of the results, which is an advantage over other spatial data sources with limited access or validity.

Thirdly, the developed methodological approach is flexible because the scale of the analysis and the level of detail can be adapted to specific research needs. In other words, the method can be freely modified. Such modifications may involve the number of land use categories and their nomenclature and characteristics, the level of detail of the land-use structure data acquired during a field inventory, analyses of orthophotomaps, the scale of elaboration, as well as the size of primary fields for interpolation.

Lastly, the method was developed specifically for large-scale mapping (in particular in urban areas), where land-use has to be effectively and accurately monitored to identify areas that are subjected to urbanization pressure and to plan and design public spaces that meet the users’ requirements and are consistent with sustainable development principles.

Therefore, the novelty is that the method can be easily adapted to the needs of users, as it is universal, highly valid, flexible, modifiable, accurate, and it can be applied to process publicly available and free sources of spatial data. The method is relatively simple, which is an additional advantage, but it requires a field inventory, which is a laborious and time-consuming procedure and the generation of maps in dedicated GIS software.

## 2. Materials and Methods

### 2.1. Designing a Methodological Approach to High-Precision Land-Use Analysis for Large-Scale Mapping

This section presents the methodological assumptions and procedure of the study. The research method developed as a result of the study is extensively characterized in the Results section. The proposed methodological approach to high-precision land-use analysis for large-scale mapping in urbanized areas was developed in several steps. An empirical study involving qualitative (desk research) and quantitative analyses (spatial and statistical analyses) was conducted to achieve the main research objective and validate the research hypothesis. In the first step, the literature on the methods and sources of data applied in land-used mapping was reviewed. The results were used to select a method that was suited to the adopted research assumptions. The available sources of spatial data were analyzed, and their suitability (validity, availability, coverage, accuracy and level of detail) for assessing land-use structure in the context of large-scale mapping was evaluated. Data sources that are publicly available and cover the entire world were selected. Land-use categories in urban space were classified. Land-use categories were defined and classified based on a review of the literature and legal regulations to generate sufficiently detailed spatial functional groups for the analysis. The main emphasis was placed on built-up and urban areas that undergo dynamic changes as a result of progressive anthropogenic pressure. In the next step, data were selected and prepared for a high-precision land-use analysis. Data were processed with the use of cartographic methods, including an interpolation algorithm, in GIS software. The selection of the appropriate methods, data sources and land-use types supported the development of a universal methodology for high-precision land-use analysis in the large-scale mapping of urbanized areas. A land-use map depicting the distribution of various land-use types in the analyzed space and maps presenting the intensity and concentration of the identified land-use types were developed in the proposed approach. The subsequent research steps of the proposed methodological approach are presented in the Results section, as they are the result of the stated goal of creating an analytical procedure and testing it. A workflow has been developed to enable its application by other researchers.

### 2.2. Research Area

Sufficiently detailed results are essential in large-scale research, including in analyses of small and medium-sized towns, districts and suburban areas. The effectiveness of the designed method was tested in the Polish town of Ostróda. The choice of location for testing the proposed method was motivated by the fact that medium-sized towns, in particular built-up areas and urban areas, are characterized by considerable diversification and fragmentation of the land-use structure. Moreover, urban areas are prone to urbanization processes, and they have a well-developed settlement structure. The selected research area features numerous land-use categories, and the fragmentation of land-use categories supports spatial analyses with the use of the proposed research method.

**Figure 1:**
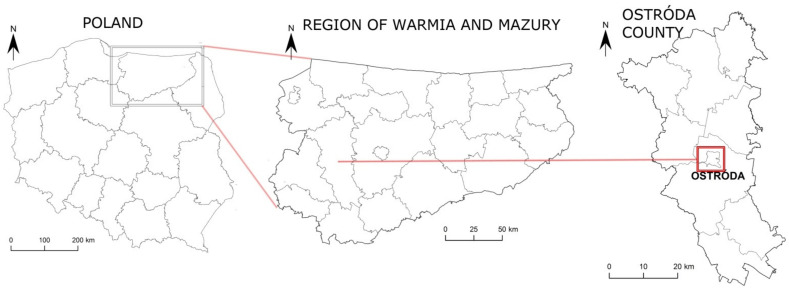
Location of the town of Ostróda in Poland, the Region of Warmia and Mazury and Ostróda county.

The analyzed urban area was the town of Ostróda in Ostróda county in the western part of the Region of Warmia and Mazury (Figure 1). The town has an area of 14.15 km^2^ and a population of 32,996 [17]. Ostróda has well-developed tourist, business, and service sectors relative to other towns and cities in the Region of Warmia and Mazury.

Geographic location and topographic features exert significant effects on urban planning and the area occupied by cities. The significance of natural features is gradually decreasing, but natural elements have always played and continue to play an important role in Ostróda. Ostróda has a diverse landscape that features glacial lakes and outwash plains that had been formed by meltwater from glaciers (sandurs). A varied landscape contributes to the town’s attractiveness and promotes the development of tourism and recreation. However, the abundance of lakes is an obstacle to continued urban development. The presence of natural barriers influences the distribution of developed areas in Ostróda [16,18].

## 3. Results

### 3.1. Desk Research

Desk research involved a review of the literature concerning the methods and data sources for analyzing the land-use structure and the classification of land-use categories in urban space. The results were used to formulate conclusions about the studied problem and to select the appropriate methods, sources, and land-use classifications for a detailed analysis of land-use structure in line with the adopted assumptions.

#### 3.1.1. Approaches to Land-Use Analysis

Various tools and methods are deployed in analyses of land-use structure in urbanized areas, including spatial analyses in GIS software, field inventories, geo-surveys, geolocation methods, and data sources supporting analyses of the land-use structure [12,13,14,15,16,19,20,21,22]. For example, an innovative combination of pixel- and object-based classification techniques and analyses of multi-temporal Landsat images in GIS software has been applied to generate land cover maps [23]. Orthophoto maps, field inventory data, and Google Earth images were used for this purpose. The study demonstrated that high-precision maps could be generated based exclusively on free multi-temporal satellite images. Machine learning algorithms promote the generation of land-use/land cover data based on satellite images [24,25,26,27,28]. Cloud computing platforms such as Google Earth Engine enable semi-automatic analyses of urban land-based on remote sensing data acquired from Sentinel-2 and Landsat satellites [29]. These platforms and learning algorithms can be applied in land-use/land cover mapping [30]. GIS tools support the integration of data from various sources, and they can be used to perform comprehensive analyses, including visualization, calculation of surface area, generation of own databases, selection of indicated objects, searching for locations and conducting network or spatial analyses. Both commercial software (such as ArcGIS developed by ESRI) and open-source programs (such as QGIS) have been used in land-use analyses [31,32,33].

As can be seen from the above review, the various approaches to land-use analysis include both different methods, tools and use different data sources. However, they have certain limitations, including possible difficulties arising from data availability (e.g., cost of acquiring data, timeliness of data, data availability only for selected time periods, incomplete coverage of the area with the necessary data, top-down land use classification applied in a manner inconsistent with the researcher’s expectations and goals, insufficient data detail, especially for areas presented on a large scale, and data fragmentation); the availability and cost of the software/hardware needed to conduct the analyses (including the ability to use it); possible difficulties with the ability to apply a particular survey method (for example, due to lack of skills, cost, lack of data availability, or time-consuming), as well as limitations due to the inability to ensure replicability of studies using the chosen method in different areas due to lack of data or lack of access to appropriate software. Importantly, many of the existing approaches do not allow for any modification to suit the needs of a particular survey (e.g., by modifying land use categories, adjusting data detail and timeliness, etc.).

The described tools enable automatic, semi-automatic or manual land-use/land-cover mapping. However, fully automated data processing is not always recommended or sufficient at a given level of analytical precision. In many cases, human knowledge and skills are essential to obtain accurate and satisfactory results. Taking this into account, there is a need for a universal approach that includes accurate methods for monitoring changes in a rapidly changing urban environment, where the detail and accuracy of the analysis are important, and where it can be modified according to research needs. Due to the limitations of the existing methods, as well as to solve the problems related to the lack of up-to-date and detailed land use maps, the authors propose a new universal methodological approach to land use structure monitoring as an effective analytical tool that is based on available and up-to-date spatial data sources and a relatively simple survey method that can be modified according to the research needs.

A review of methodological approaches and their applicability for high-precision land-use analyses prompted the authors to select and modify the cartographic inventory approach proposed by Matczak [34]. Matczak’s approach was modified, which provided a basis for the new method developed by the authors. Studies on land use structure carried out by Matczak produced highly satisfactory results in the past because it supported the creation of a land-use map presenting 10 main categories in city space. Matczak’s research method consisted of the developed town map being covered with a polygon grid (in the shape of squares). The analyses of particular land-use categories were conducted within each square, which supported the presentation of the land-use structure and the concentration and intensity of various land-use types in space. Our method uses elements of Matczak’s method. However, it is modified to make it flexible and versatile, as described in the results section.

#### 3.1.2. Data Sources for Land-Use Analysis

The results of land-use studies are highly influenced by the quality of data from various sources. Advanced remote sensing techniques and the growing availability of satellite images that facilitate land-use/land-cover mapping play an important role in land-use planning and land management [24,35,36]. Data from public registers, surveys, geo-surveys (social participation projects), points-of-interest (POI), social media, geotagged images and mobile data are used in addition to remote sensing data [21,22,37,38,39,40]. In recent years, landscape data acquired from remotely sensed images as well as building data have been most commonly used to analyze changes in land-use [41,42,43,44,45,46]. Various mapping services, such as Yahoo Maps, Bing Map, Open Street Maps (including OSM LandUse Landcover), and their national equivalents, are also very popular and constitute valuable sources of data in land-use analyses [47,48,49,50]. The available sources of data for land-use analyses are not always valid; they may cover only selected time intervals; data may be only partly available or unavailable, inaccurate, incomplete, available for a fee, and maybe excessively detailed or excessively generalized [5,51,52,53]. As a result, the repeatability of the results can be compromised in different regions [23].

The selected data sources had to be valid, widely available, universal and free of charge, and they had to provide full (not fragmentary) coverage of the studied area. Based on the adopted assumptions, the selected sources of data should support analyses of various locations around the world. It was assumed that field inventory data and orthophoto maps would be the optimal sources of spatial data. Photogrammetric data (orthophoto maps) and field inventory data characterized by high availability, relevance, ease of collection, and universality were used in the study. A similar approach was used by Bui and Musci, who relied on satellite images and field inventory data [23].

Orthophoto maps are reliable sources of information about land cover and land use. Orthophoto maps are available for almost all areas in the world and fields of study, and the relevant field inventory can be conducted almost everywhere. Moreover, the indicated data sources are sufficiently detailed, can be adapted to research needs, support the independent determination of land fragmentation in different land-use categories, and the level of generalization. The orthophoto maps used in this study had a field resolution of 25 cm (pixel size −0.25 m). The field resolution of modern orthophoto maps can be even higher (10 cm). As a result, objects can be identified with very high accuracy.

The quality, accuracy, and volume of data collected with the use of remote sensing methods are often superior to field inventory data. Due to the multitude of land-use categories in urban areas, different land-use types cannot be identified based solely on overhead photogrammetric data. Therefore, accurate identification of land-use types requires data that are collected directly in the field. For this reason, field inventories play a significant role in research, and they are suited for generating valid and accurate information about the evaluated space. These data support highly detailed and precise visualizations of land management. During a field inventory, the land-use structure is examined directly in the field, and the resulting observations are presented graphically on a map. A field inventory is the most up-to-date source of information because of the level of detail that is most appropriate for the conducted research; the time and place of the inventory are defined by the researcher. The errors and limitations associated with the existing data sources (orthophoto maps, cadastral data, and data from other spatial databases), including limited accessibility, invalidity, insufficient detail, and land-cover types that do not meet research needs, can be eliminated in a field inventory. However, field inventories also have certain limitations. A field inventory generates data that are most valid, detailed, and accurate, but this approach is highly laborious, time-consuming, and it requires visits to the study site. Therefore, field inventories are best suited for analyses of small areas (small towns, villages, residential estates, urban districts, suburban areas). In studies focusing on large areas, the process of conducting field inventories and generating maps becomes highly complex.

In the present study, the field inventory involved two interconnected procedures, i.e., a field survey and field mapping, to determine the land-use structure in the studied site. The inventory was conducted with the use of a topographic map in 1:10,000 scale [54]. The conducted research consisted of direct observation/inspection of the area, including the preparation of photographic documentation (field survey) and collected information about the location of different land-use types and marking them on the map (field mapping). For best results, cartographic data should be visualized with the use of various tools, such as paper maps and mobile devices [55]. The collected information is then saved as vector data in GIS software.

#### 3.1.3. Classification of Land-Use Categories in Urban Space

Various land-use classification criteria characterized by different levels of detail are available in international and domestic sources [46,56,57,58,59,60]. General sources include the classification proposed by Pei et al. [61], who distinguished five categories of land-use according to aggregated mobile phone data covering residential, business, commercial areas, open space and others. Huang et al. [46] relied on a land-use taxonomy predefined by the Land Administration Law of the People’s Republic of China (GBT 21010-2017). Based on the adopted methodology, nine of the twelve land-use categories (cultivated land, garden land, woodland, grassland, commercial land, industrial land, residential land, public management, and public service land, special land, transportation land, water and water conservancy facilities, and other lands) were selected as the most important categories for the final urban land-use taxonomy [46]. More detailed sources include the CORINE Land Cover database [57], which contains 44 land-cover classes grouped in a three-level hierarchy (the five main categories are artificial surfaces, agricultural areas, forests and semi-natural areas, wetlands, and water bodies), and the Polish cadaster [62] with 26 land-use classes in six land-use categories (agricultural land, forest areas, built-up areas, and urban areas, ecological sites, land underwater, other areas). However, the Polish Database of Topographic Objects (DBTO10k) contains data at three levels of detail. The first level includes eight categories, the second–57 classes, and the third consists of 286 types [63]. The availability of data characterized by various levels of detail supports the selection of the optimal classification scale for spatial analysis.

The classification of land-use categories should be flexibly adapted (detailed or generalized) to the studied area and various diagnostic needs, such as functionality analyses. In the classification process, the required level of detail was determined for the needs of large-scale mapping of the land-use structure in urban areas. The most important land-use categories were defined to eliminate data that are excessively detailed (which can compromise comparisons between a larger number of research areas and lead to excessive fragmentation of various categories of the analyzed data) and excessively generalized (which can undermine the reliability of the results; for example, when the analyzed area encompasses various land-use types, such as industry and services). These categories also enable analyses and evaluations of the location of different functions in urban space to create a user-friendly environment.

For the needs of this study and to validate the proposed method, the land-use classes/types in the Polish cadaster, the Database of Topographic Objects (DBTO10k), the CORINE Land Cover repository, as well as other databases presented in the literature [34,56,57,62,63], were identified. The identified land-use types were then modified to develop a universal classification system. The main land-use categories in built-up areas and urban areas were also determined. Six land-use categories were identified in developed areas: residential areas, services, transportation, industrial and storage facilities, public green spaces and recreational areas, other developed areas. In undeveloped (open) areas, four land-use categories were identified: agricultural land, forests, water bodies and streams, and other undeveloped areas. The following criteria were used to classify land-use types in developed urban areas (Table 1).

A similar approach was deployed by Huang et al. [46], who identified the following land-use types: commercial, residential, educational, natural, civic, transport, industrial, agricultural, other.

The presented criteria for the classification of land-use types in developed urban areas can be applied to various spatial units, including towns, cities, residential areas, districts, and suburbs. The adopted classification can be used not only in Poland, but also in urban areas in other countries.

### 3.2. A Methodological Approach to High-Precision Land-Use Analysis for Large-Scale Mapping

For the needs of the methodological approach to high-precision land-use analysis for large-scale mapping, Matczak’s approach [34] was modified by introducing a field inventory and orthophoto maps, and geostatistical processing was adapted to large-scale requirements based on the possibilities offered by GIS and the available sources of spatial data. Data were processed in ArcGIS software which supports complex spatial analyses.

**Figure 2:**
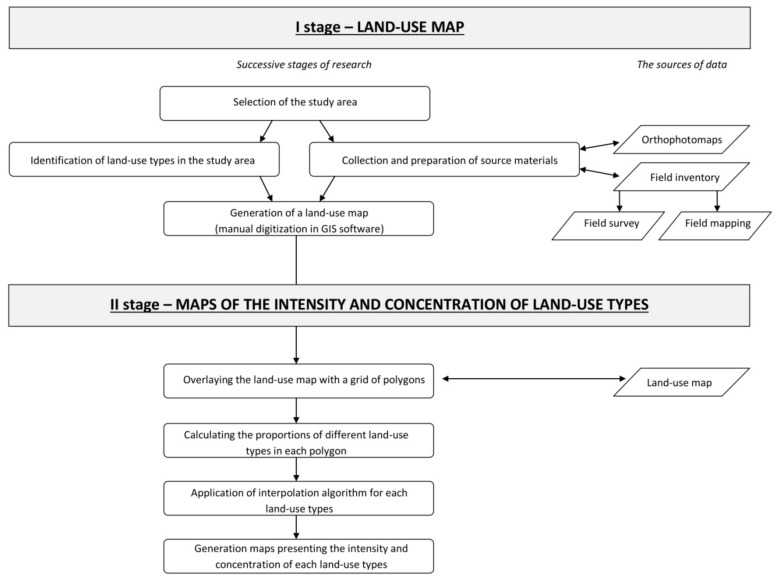
Successive stages of research for high-precision land-use analyses in the context of large-scale mapping.

The successive stages of research involving the proposed method and the process of generating maps based on the described approach are presented in Figure 2.

The proposed method contains two main research stages. A map depicting the distribution of different land-use types in the analyzed space is generated in the first stage. In the second stage, the generated map is used to develop maps of the intensity and concentration of various land-use types. Therefore, the proposed method offers two approaches to visualizing the evaluated phenomena.

#### 3.2.1. Generation of a Land-Use Map

In the first stage of the research (creating a land-use map), photogrammetric data (orthophoto maps) and field inventory data characterized by high availability, relevance, ease of collection, and universality were used. The orthophoto maps used in this study had been developed in 2017 and had a field resolution of 25 cm (pixel size −0.25 m). A field inventory of the studied area was conducted over a period of two months in 2017.

A map illustrating the land-use structure in the examined town was generated in the next step of the study. The orthophoto maps (in Web Map Tile Service (WMTS) format) and the materials acquired during the field inventory (vector data) formed separate layers, and they were used to manually digitize polygons in GIS software. Digitized land-use types were assigned locations and attributes. This approach to collecting data and generating land-use maps is laborious and time-consuming, but it supports accurate and up-to-date presentations of land-use structures.

#### 3.2.2. Generation of Maps of the Intensity and Concentration of the Identified Land-Use Types

The developed land-use structure map was used to generate maps of the intensity and concentration of the identified land-use types. In the first step of the process, the studied area (land-use structure map) was overlaid with a grid of primary fields (grid of polygons), and the identified land-use types were analyzed inside each field. Grids of primary fields (squares, hexagons, etc.) support detailed analyses of spatial phenomena [34,64,65]. The size of primary fields for successive analyses can be modified, subject to the required level of detail. It was assumed that a 500 × 500 m grid and smaller grids (e.g., 100 × 100 m, 250 × 250 m, 400 × 400 m) would provide the required level of detail for analyses of land-use structure in large-scale mapping [16,66]. Smaller grids of primary fields generate more detailed results, whereas larger grids produce more generalized results; therefore, the level of detail that is necessary to achieve the research goals has to be set at the beginning of the process.

**Figure 3:**
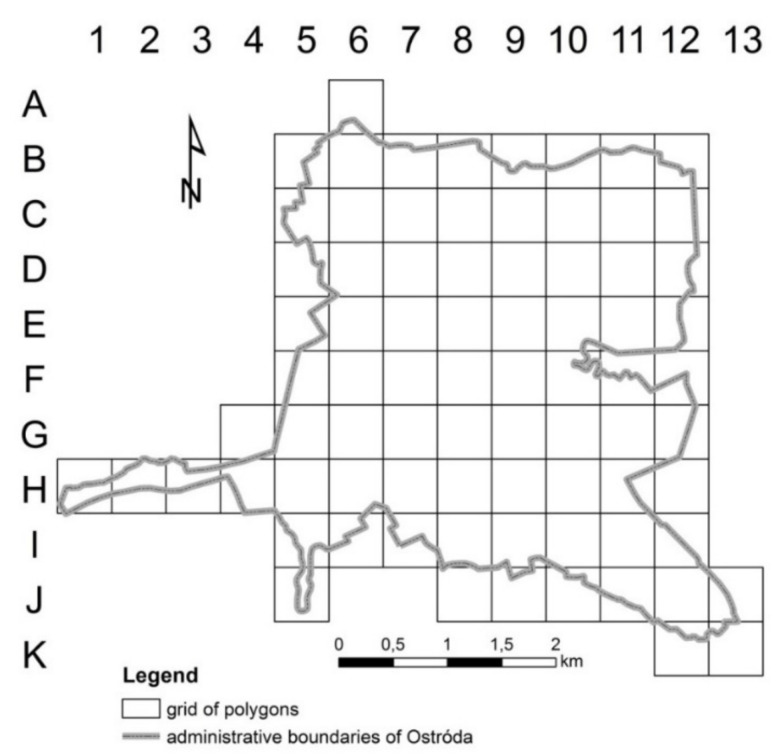
The planned grid of polygons within the Ostróda city area. Source: own elaboration.

A 500 × 500 m grid of primary fields was adopted for the needs of the study as a grid that would provide the required level of detail for presenting the results of the proposed research method. The contour map of the investigated town was overlaid with a grid of 79 polygons (79 primary fields) measuring 500 × 500 m each (Figure 3). The size of the polygons supported a detailed analysis of the evaluated area. Polygons were assigned unique names (A6, B5, etc.–Figure 3). The area occupied by every land-use type was calculated inside every polygon, and a database was created.

The studied town was divided into primary fields, which facilitated statistical analyses. The proportions of different land-use categories were determined in each field, and maximum, minimum and mean values and standard deviation were calculated. The dominant land-use types were identified in each field. This approach not only offers an alternative method of visualizing spatial information about the land-use structure but also facilitates statistical analyses by generating a grid of primary fields, which cannot be achieved with the use of standard land-use maps.

An interpolation algorithm was deployed in the next step to generate maps of the intensity and concentration of the identified land-use types. Data were interpolated based on a grid of 79 polygons (primary fields). The central point in each polygon was assigned a value denoting the proportion of every land-use type in the total polygon area.

Interpolation methods are used to present the analyzed phenomena in a continuous manner. The parameters measured in specific points are converted into continuous surface data to illustrate the spatial distribution of the studied phenomena [67,68]. Interpolation methods are applied in analyses of land-use structures because the land cover is a phenomenon that occurs throughout the entire evaluated space. It should be noted that isolines do not accurately represent real-life phenomena. Interpolated boundaries are “smoothed” relative to real areas.

Various interpolation methods have been developed, including spline, kriging, and IDW [68]. Spline interpolation was selected to test the applicability of the proposed method in the studied area because it produced the most satisfactory visual results. The proposed methodological approach is flexible; therefore, other interpolation methods that are best suited to research needs can be selected.

Data were interpolated with the use of spline interpolation in ArcGIS software. This deterministic interpolation method produces continuous surfaces and minimizes the “roughness” of real-life data [69,70]. Spline interpolation supports the generation of smooth surfaces with a large number of data, and it produces satisfactory results in irregular surfaces [71]. The spline algorithm interpolates surfaces based on Formula [72] (1):

(1) $$S\left(x,y\right)=T\left(x,y\right)+\sum_{j=l}^{N}\lambda_{j}\;R\left(r_{j}\right)\;$$

where:

j = 1, 2,…, N,
N—number of points,
λj—coefficients determined by solving a system of linear equations,
rj—distance from point (x, y) to the j-th point.

The value of T(x, y) and R(r) varies subject to the selected form of spline interpolation.

Data were interpolated separately for each of the six land-use types in developed areas. Data processed by the interpolation algorithm were used to generate maps of the intensity and concentration of different land-use types. Areas with a high concentration of various land-use types can be clearly identified in the resulting maps. Areas characterized by the highest concentration and intensity of specific land-use types that were distinctly separated from the surrounding space were regarded as clusters.

### 3.3. Testing the Approach—A Case Study of Ostróda

The implementation of the research method supported the creation of a database containing detailed information about the land-use structure.

**Figure 4:**
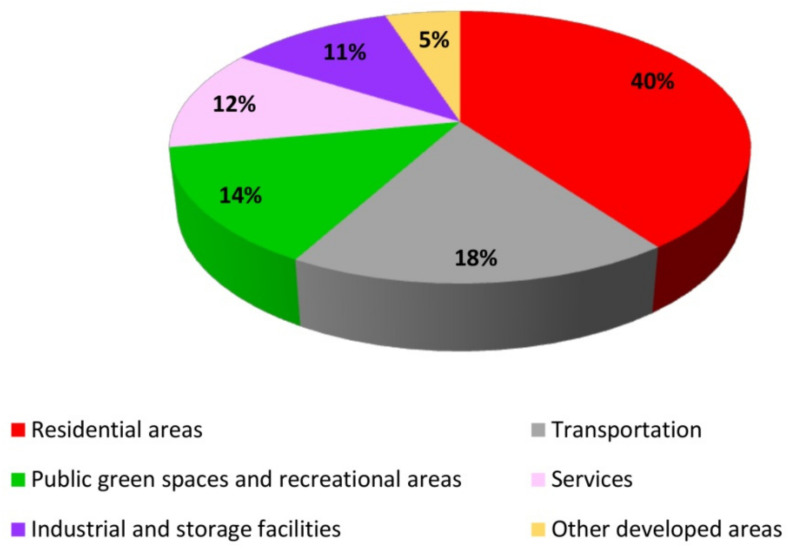
Land-use structure in developed areas in the town of Ostróda in 2017.

**Figure 5:**
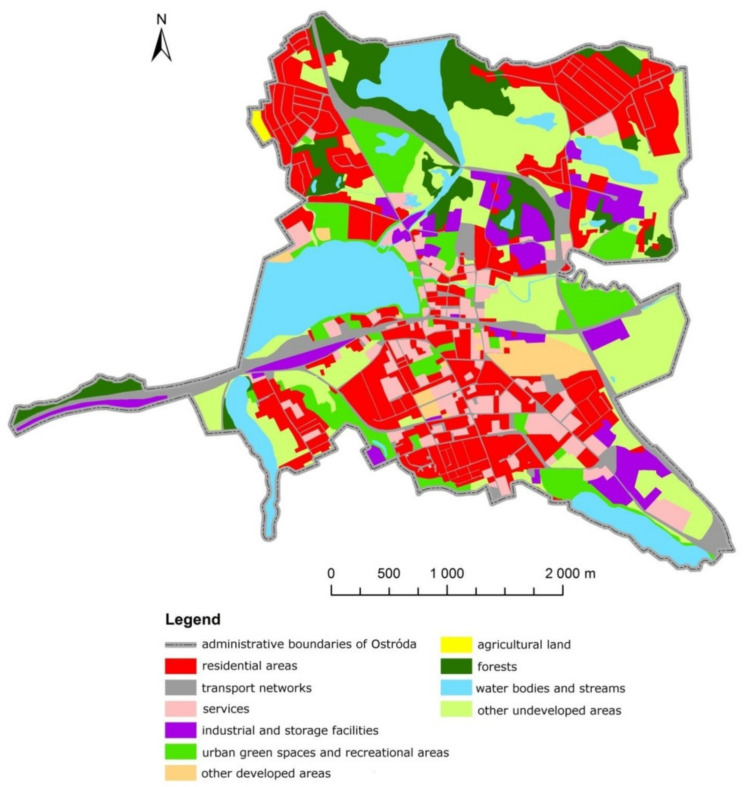
Land-use structure in the town of Ostróda in 2017.

**Figure 6:**
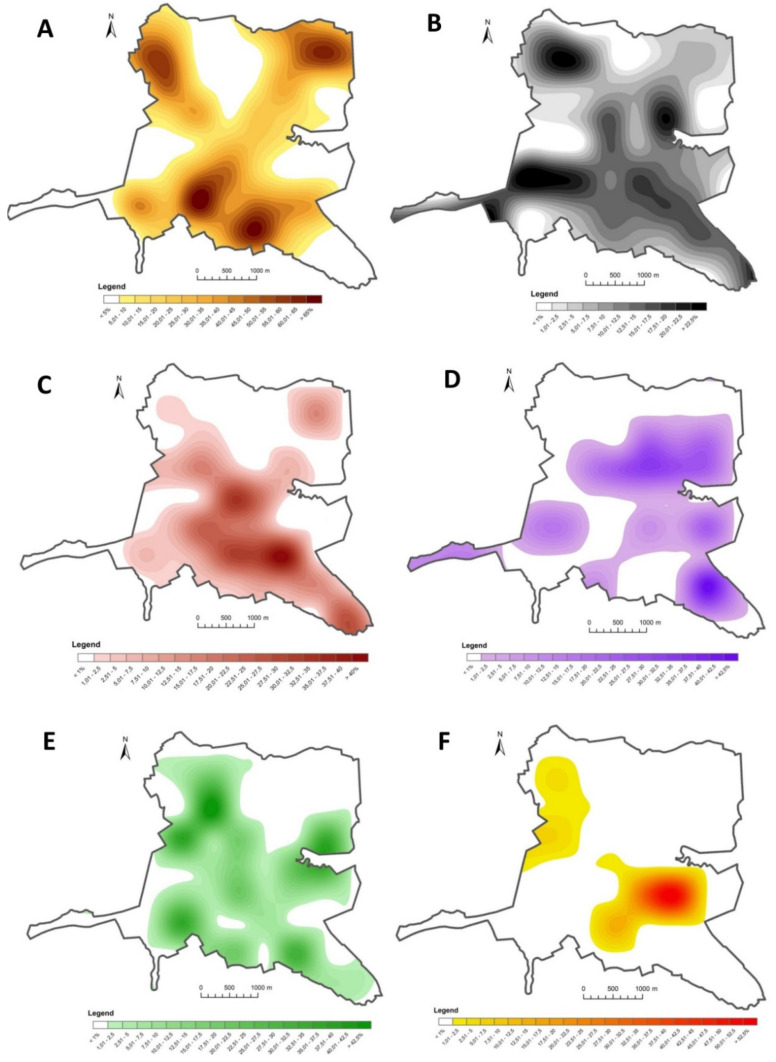
Intensity and concentration of residential areas (**A**), transport networks (**B**), services (**C**), industrial and storage facilities (**D**), urban green spaces and recreational areas (**E**), other developed areas (**F**).

The analysis supported an accurate determination of land development in the town of Ostróda (Figure 4). The collected materials and the obtained results were used to describe various land-use categories in urban space. The presented results were obtained with the use of a generated land-use map covering the entire town (Figure 5) as well as maps presenting the concentration and spatial distribution of different land-use categories (Figure 6). Detailed analyses focused on built-up areas (residential areas, services, transportation, industrial and storage facilities, public green spaces and recreational areas, other developed areas) which undergo dynamic change.

In 2017, developed areas accounted for approximately 60% of the investigated urban space. In these areas, the natural environment has been considerably transformed, and the existing buildings, structures, and land-use types are characteristic of urban development. Residential areas (40%) were the predominant land-use type (Figure 4), followed by transport routes (18%), public green spaces, and recreational areas (14%). Services accounted for 12% of urban space. Industrial and storage facilities occupied more than 11%, and other developed areas–more than 5% of urban space. The presented land-use structure indicates that the residential function plays a predominant role, and transportation plays a very important role in Ostróda.

Residential areas are predominant in Ostróda, and they occupy 40% of urban space. The distribution and concentration of residential areas in Ostróda are linked with population and environmental conditions. Five distinctive residential clusters were identified in Ostróda–two in the northern part and three in the southern part of the town (Figure 6A). The distribution of residential clusters resembles the letter X, and higher levels of development are noted in the southern part of the town. Due to topographic features and local landform, there is a prevalence of single-family homes in the north, whereas apartment buildings dominate in the south. Two distinctive residential clusters were identified in the northern part of Ostróda, where residential buildings occupied more than 55% of the polygon area. These clusters are characterized by dense single-story and double-story development. The southern part of Ostróda has a moraine landform, and it features mostly apartment buildings that formed three residential clusters. The proportion of residential development exceeds 50% in the first cluster (south-western Ostróda) and 65% in the remaining two clusters (southern Ostróda).

Transport networks are an indispensable feature of developed areas in cities, and their distribution and coverage are determined by a city’s spatial layout, geographic location as well as location relative to other settlements. In Ostróda, transport networks represent the second most prevalent type of land-use after residential areas, and they form the backbone of the urban fabric. The road system is characterized by considerable fragmentation (Figure 6B). Ostróda is an important transport hub in the local and national road network. Two major (national) roads and a railway line intersect the town. The western part of the town features a railway station, a bus station, and railway sidings which account for more than 35% of the land occupied by transport networks. Transport networks and transit traffic facilities, including petrol stations and large transport hubs, form two clusters in the north and north-east (up to 25% coverage).

The size and utilization of service areas are influenced by a city’s administrative functions, local demand for support facilities, historical factors, and the number of inhabitants. Service areas generally coexist with other land-use types, mostly residential (where service outlets occupy the ground floor of apartment buildings); therefore, only services that exist independently or play a dominant role relative to other urban functions were analyzed in the study. It should be noted that these types of services account for only a certain proportion of service outlets in an urban area. Services were generally distributed (Figure 6C) along a line stretching between the north-western and the south-eastern parts of the town, and the highest number of service outlets was found in central and southern Ostróda. Services had a similar distribution pattern to residential areas. Services formed three clusters in central and south-eastern parts of the town, and they accounted for 35% of the polygon area. Dispersed service outlets, mostly in residential areas (large-area retail outlets, educational facilities), were also located in the north-eastern, north-western, and south-western parts of the town. The area occupied by services varied subject to outlet type. Some service outlets had a small area and were used intensively, whereas others occupied a large area and were characterized by less intensive use.

Industrial and storage facilities formed seven clusters in Ostróda (Figure 6D). Most clusters were found in the eastern part of the town, where they occupied 45% of the polygon area. Industrial and storage facilities were generally located in the vicinity of major transit routes. Two high-density clusters in the northern and south-eastern parts of Ostróda were linked by two clusters where industrial facilities were less densely distributed. Two clusters in the western part of the town and one cluster in the southern part of the town were also identified. Industrial and storage facilities had a different distribution pattern than residential areas. The largest industrial cluster comprising construction companies, factories, and shipbuilding plants, was found in the northern part of the town. Production plants were concentrated in eastern Ostróda for environmental reasons: due to a predominance of westerly winds, pollution is carried in the eastern direction outside the town’s administrative boundaries. The concentration of industrial and storage facilities was highest in south-eastern Ostróda, where this type of land-use occupied more than 42.5% of the polygon area. Industrial sites were separated from residential areas and services by green belts and undeveloped areas.

Public green spaces and recreational areas are developed areas that are intended for passive and active recreation. In Ostróda, urban green spaces and recreational areas occupied 1.12 km^2^ and accounted for 13.56% of developed areas and 7.92% of the town’s territory. This land-use type had a higher share of land cover than services and industrial and storage facilities, which has positive environmental implications. In Ostróda, the distribution of public green spaces is influenced by the location of water bodies and subglacial tunnel valleys. Green areas are an inseparable element of the largest lakes in the town. They improve Ostróda’s aesthetic appeal and create numerous recreational options. In the studied town, urban green spaces formed eight clusters with different intensities of use (Figure 6E). These clusters formed an elongated strip that stretched from the north-western to the south-eastern part of the town. A high concentration of urban green spaces, including allotment gardens, was noted in eastern Ostróda (>40% of polygon area) in the vicinity of the glacial valley of Drwęca River. A municipal beach, sports, and recreational facilities, and allotment gardens formed a large cluster in north-western Ostróda (>45% of polygon area). Allotment gardens accounted for nearly 50% of urban green spaces and recreational areas in the town; they were accessible only to owners.

Other developed areas included former military grounds, construction sites, privately owned developed land that is not used for residential purposes, services, industrial or storage purposes, as well as developed areas that have been abandoned. These areas are most rapidly transformed to serve new functions. Other developed areas occupied 0.44 km^2^; they accounted for 3.11% of the town’s territory and 5.33% of the total developed area in Ostróda. These areas formed two clusters in north-western and eastern Ostróda (Figure 6F). The cluster in the eastern part of the town was composed of former military grounds that were gradually transformed into residential areas. This land-use category accounted for 55% of the polygon area. Military facilities (bases, installations, stations) and entire military units were closed down and converted to other functions after the restructuring of the Polish armed forces.

The distribution of developed areas in Ostróda is determined mainly by environmental features, historical factors, spatial attributes, and local planning decisions. In the analyzed town, land development is inhibited mainly by natural barriers (water bodies, glacial valleys, protected areas) that are nearly impossible to overcome.

The grid of primary fields supported the determination of minimum, maximum and mean values as well as the standard deviation for each land-use category in developed areas (Table 2).

The minimum proportion of each land-use category in a primary field is zero, which indicates that a given land-use category was not encountered in at least one primary field. The greatest variation was noted in residential areas, and depending on their concentration in a primary field, the proportion of residential areas ranged from 0% to nearly 67%. Transportation services had the smallest share of the primary fields (from 0% to nearly 26%). Despite the small total area occupied by other developed areas (urban space accounted for more than 5% in only 15 out of 79 primary fields), their proportion in primary fields ranged from 0% to nearly 55%, which points to a high concentration of other types of developed areas in the analyzed town. Transportation services and residential areas were extensive and occupied 66 and 54 out of the 79 primary fields, respectively. The standard deviation for transportation services and residential areas was similar to the respective mean values (7.14 vs. a mean value of 7.45, and 18.95 vs. a mean value of 16.73, respectively). Clear differences were noted between standard deviation and the mean values of industrial and storage facilities, public green spaces and recreational areas, as well as other developed areas. In the first two cases, the standard deviation exceeded the mean value two-fold, whereas the standard deviation of other developed areas exceeded the mean value four-fold, which points to considerable differences in the proportions of these land-use types in primary fields.

The results obtained with the use of the proposed method not only supported the determination of the present land-use structure in the town but were also used to identify areas with the highest and lowest intensity and concentration of specific land-cover types. Maps presenting the intensity and concentration of various land-use types offer a different analytical perspective than maps that present the distribution of different land-use types in space. The distribution of land-cover types in urban areas provides only indirect information about their concentration and intensity, and the relevant data are generally used to determine that such land-use types exist in a given area. The application of interpolation methods in analyses of land-use structure creates new opportunities for assessing the intensity and concentration of different types of land cover in urban space, and the proposed methodological approach and interpolation algorithm facilitate statistical analyses.

## 4. Discussion

Other sources of spatial data that could be potentially applied in the research process were considered to verify the research hypothesis. The aim of the comparison was to determine whether other data sources were sufficiently precise, universal, topical, and useful for analyzing the land-use structure and diagnosing spatial development (in particular in the context of large-scale mapping).

The proposed research method relied on orthophoto maps and field inventory data. Other data sources were also reviewed on the assumption that a universal research method for large-scale mapping should rely on publicly available, free, comprehensive, and valid data that provide full (not fragmentary) coverage of the studied area. The following spatial databases were considered: the cadaster, Urban Atlas, Database of Topographic Objects (DBTO10k), OpenStreetMap (OSM) LandUse Landcover, and the CORINE Land Cover (Table 3). These databases are fully developed repositories of information about land-use/land cover; therefore, additional (and often laborious) efforts are not required to acquire the necessary data. Other spatial databases also exist, but they are thematic databases (e.g., riparian zones, coastal zones) and therefore were excluded from the analysis.

Cadastral data are the most accurate, detailed and up-to-date sources of information on land-use in large scale mapping [73,74]. However, cadastral data are not always valid, and they are not always available in all countries, or they do not cover the investigated area [75]. In many countries, attempts are being made to develop cadastral databases, but detailed data repositories are not available in other countries [76]. Data for large-scale analyses of land-use (such as CORINE Land Cover data) are characterized by low precision and considerable generalization [77]. The CORINE Land Cover uses a minimum mapping unit of 25 hectares for aerial phenomena and a minimum width of 100 m for linear phenomena [78]. The fact that the compared data cover only specific time intervals is yet another limitation. More detailed databases, such as the Polish Database of Topographic Objects (DBTO10k), cover only Poland, and different regions are updated at various time intervals, which prevents a reliable comparison of regions, are not always up-to-date, and they do not support comparisons within a longer time interval. Moreover, the availability of such data is often restricted to specific countries, such as Poland [60]. The Urban Atlas is a valuable source of data on the land-use structure [79]. This resource covers the entire European continent, but it is limited to functional urban areas (FUAs). In 2018, 696 FUAs were covered by the Urban Atlas, which implies that data relating to small and medium-sized towns outside the FUAs are not available. OSM LandUse Landcover data include a relatively small number of land-use types in urban areas (for example, industrial, commercial, and transport units were classified into one type of land-use, which at the level of detailed analysis may be insufficient differentiation; in addition, in 2017 data for the studied town were not available) [50].

The proposed research methodology is characterized by high validity, a high level of detail, and accuracy. The suggested sources of data (field inventory and orthophoto maps) guarantee the validity of the obtained results; therefore, the described method has a considerable advantage over approaches that rely on spatial data with limited availability and validity, and that are available for a fee and are characterized by limited spatial coverage. A field inventory also eliminates the problem of incomplete coverage and generates the most suitable and up-to-date information for research purposes. During a field inventory, different land-use types can be selected and modified to suit specific research needs.

## 5. Conclusions

The results of this study validated the research hypothesis postulating that the research methodology for generating valid, accurate, and customized land-use maps based on an interpolation algorithm, widely available photogrammetric data, and field inventory data is a precise, universal and useful tool for diagnosing spatial development, in particular in the context of large-scale mapping. The proposed research methodology is highly universal, and it can be applied to investigate land-use structures in small towns, medium-sized and large cities, urban agglomerations, suburban and rural areas around the world. The proposed method offers two approaches to visualizing the analyzed phenomenon in space. The fact that the last stage of the land-use structure analysis can be freely defined is yet another advantage of the described method. Depending on research needs, the analysis can be terminated after the generation of a land-use map, and successive steps involving the interpolation algorithm and the generation of maps of the intensity and concentration of different land-use types can be abandoned. The interpolation algorithm can be applied only to selected land-use types in line with research assumptions and goals. The research procedure can thus be terminated at any point without compromising the results.

The proposed methodology is also highly flexible, and it can be modified for specific research needs. Firstly, the researcher can adapt the land-use classification to specific research purposes (by selecting and defining other land-use types or applying different nomenclature). Secondly, the scale of the analysis and the level of detail of land-use structure data can be modified during a field inventory. In addition, various interpolation methods (IDW, kriging, spline) can be applied in the interpolation algorithm, and the size of primary fields can be adapted to the needs of the interpolation process.

The main strengths of the proposed methodological approach are its universal character, high validity, flexibility, modifiability, accuracy, and suitability for processing data that are available to the public at no additional charge. The described method is also relatively simple to use.

Despite numerous advantages, the proposed method has certain limitations. Firstly, it requires a field inventory which is a laborious and time-consuming process, as well as dedicated GIS software for generating maps. In studies covering large areas (for example, urban agglomerations), such analyses are even more complex and laborious. Secondly, a field inventory is a time-consuming procedure that requires a visit to the study site, which could pose a significant challenge in analyses of foreign locations or sites that are situated remotely from the researcher’s place of residence. In addition, the process of customizing classifications of land-use categories can be subjective.

It should also be noted that the proposed method can be applied to generate maps based on various sources of spatial data. However, the differences between the existing sources of data and their limitations (validity, level of detail, availability, scope, type of access, nomenclature, and various land-use types) should be taken into consideration to obtain valid and reliable results.

Universal methodological approaches and widely available sources of data should be used in analyses of land-use structures to guarantee the reproducibility of the results. The specificity and complexity of the proposed method and the associated hardware and software requirements could also pose a challenge, and they could limit the repeatability of the described approach. Therefore, the quality of publicly available databases should be examined, and only databases that are most suitable for practical application should be selected for analytical purposes. Each data source applied in analyses of land-use structure should be verified because spatial data evolve rapidly and are frequently modified.

Further research is needed to develop new methods or improve the existing methods for monitoring land-use structure, in particular in urbanized areas. These efforts require universal and flexible methodological approaches. The validity and availability of spatial data sources should be continuously monitored because spatial data evolve rapidly and are frequently modified.

## Figures and Tables

**Table 1:** Criteria for classifying land-use types in urban areas.

Land-Use Type	Description
Developed Areas	
Residential areas	Areas zoned for residential construction and the accompanying functions (such as services) that complement the residential function or play a minor role relative to the main function (such as retail outlets that occupy the ground floor of apartment buildings).
Services	Commercial (retail outlets, restaurants, transport, repair shops, finance, insurance, conference services, hotels) and public services (education, health care, social services, culture, art, public administration at the central and local level, justice administration, political and social organizations). Services generally coexist with other land-use types, mostly residential (service outlets that occupy the ground floor of apartment buildings); therefore, only services that exist independently or play a dominant role relative to other functions were analyzed in the study. It should be noted that these types of services account for only a certain proportion of service outlets in an urban area.
Transportation	This category includes streets, railway tracks, squares and facilities supporting road and railway traffic, including garages, parking lots, bus depots, railway sidings, railway stations and petrol stations. Roadside greenery and green belts were also included in the analysis. Walkways in residential estates and internal roads in industrial parks and business complexes were not taken into account.
Industrial and storage facilities	This category includes industrial facilities, production plants, administration buildings in industrial plants, storage yards and warehouses, as well as technical facilities for power and gas grids. Protective green areas surrounding industrial facilities were also taken into account in the analysis.
Public green spaces and recreational areas	This category includes parks, pocket parks, allotment gardens, cemeteries, sports facilities and public beaches. Green areas that serve additional functions in other land-use categories (such as residential greenery, roadside greenery, green belts surrounding industrial facilities, sports fields and sports buildings in schools) were not taken into consideration.
Other developed areas	This category includes former military grounds, construction sites, privately owned developed land that is not used for residential purposes, services, industrial or storage purposes, as well as developed areas that have been abandoned. These areas are most rapidly transformed to serve new functions.
Undeveloped (open) areas	
Agricultural land	This category includes arable land, which is cultivated in agriculture and horticulture, as well as fallow land.
Forests	This category comprises forests, i.e., land with a compact structure that is covered by forest vegetation (trees, shrubs, groundcover), is intended for forestry production, or constitutes a nature reserve or a national park, and is associated with forest management. This category also includes land covered by forest plants. This category does not include clusters of trees and shrubs in parks, cemeteries, gardens and sports fields, individual trees, and small tree clusters.
Water bodies and streams	This category covers all natural water bodies and artificial water reservoirs, including lakes, rivers, canals, watercourses, streams, ponds and man-made reservoirs.
Other undeveloped areas	This category includes undeveloped land that has not been classified in the remaining categories, such as meadows, waterlogged areas, marshes, barren land, individual trees and shrubs, and tree and shrub clusters.

**Table 2:** Basic statistical data from an analysis of primary fields.

	Max [in %]	Min [in %]	Mean	Standard Deviation	Number of Primary Fields with a Given Land-Use Category
Residential areas	66.70	0.00	16.73	18.95	54
Transportation	25.47	0.00	7.45	7.14	66
Services	40.81	0.00	5.02	9.04	34
Industrial and storage facilities	44.72	0.00	4.89	9.05	30
Public green spaces and recreational areas	47.49	0.00	5.73	10.68	36
Other developed areas	54.58	0.00	1.64	7.33	15

**Table 3:** Description of spatial data sources.

				Database			
	Cadaster	Urban Atlas	Database of Topographic Objects (DBTO10k)	OSM LandUse Landcover	CORINE Land Cover	Orthophoto Maps	Field Inventory
Coverage	National cadasters	Partial coverage in Europe (only Functional Urban Areas)	Only Poland	All European countries	All European countries	The entire world	Defined by the researcher
Availability/paid or free access	Depending on country. In Poland: Available for public viewing at no charge; available for processing for a fee.	Available to the public at no charge	Available to the public at no charge since 2020. Previously available upon request and for a fee.	Available to the public at no charge	Available to the public at no charge	Available to the public at no charge	Free, the inventory is conducted by the researcher
Validity of available data	Depending on region	2018	Depending on region (data valid for 2013–2020)	2020	2018 (based on satellite images captured in 2017)	2021 Depending on data source and region	High validity (a field inventory depicts the present land-use structure)
Update frequency	Depending on data source (updated continuously or periodically)	Every 6 years	Depending on region (every few years)	Depending on region	Every 6 years	Depending on data source and region	Data sources are updated by the researcher according to need

## Data Availability

Data is contained within the article.
